# The Mast Cell-Aryl Hydrocarbon Receptor Interplay at the Host-Microbe Interface

**DOI:** 10.1155/2018/7396136

**Published:** 2018-10-28

**Authors:** Claudio Costantini, Giorgia Renga, Vasilis Oikonomou, Giuseppe Paolicelli, Monica Borghi, Marilena Pariano, Antonella De Luca, Matteo Puccetti, Claudia Stincardini, Paolo Mosci, Andrea Bartoli, Teresa Zelante, Luigina Romani

**Affiliations:** ^1^Department of Experimental Medicine, University of Perugia, Perugia 06132, Italy; ^2^Department of Pharmaceutical Science, University of Perugia, Perugia 06132, Italy; ^3^Department of Veterinary Medicine, University of Perugia, Perugia 06132, Italy

## Abstract

Mast cells are increasingly being recognized as crucial cells in the response of the organism to environmental agents. Interestingly, the ability of mast cells to sense and respond to external cues is modulated by the microenvironment that surrounds mast cells and influences their differentiation. The scenario that is emerging unveils a delicate equilibrium that balances the effector functions of mast cells to guarantee host protection without compromising tissue homeostasis. Among the environmental components able to mold mast cells and fine-tune their effector functions, the microorganisms that colonize the human body, collectively known as microbiome, certainly play a key role. Indeed, microorganisms can regulate not only the survival, recruitment, and maturation of mast cells but also their activity by setting the threshold required for the exploitation of their different effector functions. Herein, we summarize the current knowledge about the mechanisms underlying the ability of the microorganisms to regulate mast cell physiology and discuss potential deviations that result in pathological consequences. We will discuss the pivotal role of the aryl hydrocarbon receptor in sensing the environment and shaping mast cell adaptation at the host-microbe interface.

## 1. Introduction

Mast cells (MCs) originate from hematopoietic stem cell-derived progenitors that leave the bone marrow, enter the circulation, and populate all vascularized tissues [1]. MCs phenotypically express the surface markers c-kit and Fc*ε*RI [2], both required for MC survival by binding stem cell factor (SCF) and monomeric IgE, respectively [3, 4]. Specific cell adhesion molecules and chemokine receptors selectively drive MC homing to distinct peripheral tissues [5], and lipid mediators may contribute to MC recruitment during inflammation [5]. Upon settling down in tissues, MCs terminally differentiate and become competent for the exploitation of their effector functions. Indeed, mature MCs are equipped with a plethora of effector molecules including proteases, vasodilating substances, cytokines, chemokines, and lipid mediators [6] as well as an ever increasing repertoire of receptor molecules that fine-tune their activation [7]. This deeper understanding of the molecular machinery that enables MCs to sense and respond to environmental cues is accompanied by a wider view of MC functions that goes beyond their long-recognized role in allergic responses to include protection of the host against infections and modulation of adaptive immune responses [6]. Two features are worth mentioning when contextualizing the functions of mature MCs. The first one is represented by the specific location within the peripheral tissues. Indeed, MCs strategically locate at sites exposed to the external environment, thus being able to interact with foreign agents. At the same time, the microenvironment appears to play a crucial role in shaping MC development, phenotype, and function, which results in a spatial and temporal heterogeneity and plasticity of MCs [8]. Altogether, it is becoming increasingly evident that a continuous cross-talk between MCs and the surrounding environment is crucial for the maintenance of tissue homeostasis and that a dysregulated interaction may predispose to the insurgence of inflammatory and/or allergic conditions.

The microbiome represents the entirety of microorganisms that colonize the human body. Virtually, all externally exposed surfaces harbor a variety of microbial communities [9]. The microbiome plays important functions in host tissue homeostasis and, consequently, dysbiosis, i.e., an altered state of the microbial community, is causally linked to a variety of pathological conditions [10], including tumor development [11]. A delicate cross-talk between commensal microorganisms and the immune system ensues to prevent inappropriate activation of the immune system against commensal bacteria and maintain a tolerogenic state [12]. In turn, dysbiotic changes predispose the host to the development of immunological diseases [13]. Several mechanisms have been identified in the cross-talk between the microbiome and the immune system [12, 13]. One such mechanism involves the aryl hydrocarbon receptor (AhR), a ligand-dependent transcription factor that senses both xenobiotic and endogenous ligands, including microbiota-derived factors [14, 15]. Specifically, AhR contributes to immune homeostasis with a dual role: on the one hand, it is endowed with an antimicrobial role that comes from the AhR-dependent IL-22 transcription; on the other hand, it possesses an anti-inflammatory role by mediating the differentiation of regulatory T cells (Tregs) [15]. Based on these premises, the location of MCs in close proximity to the external environment and their plasticity driven by surrounding cues make the microbiome an ideal partner in the regulation of MC physiology.

In this review, we will discuss the current knowledge on the ability of the microbiome to regulate the survival, recruitment, maturation, and function of MCs as well as potential deviations that result in pathological conditions. We will consider the molecular mechanisms that underlie the cross-talk between the microbiome and MCs with particular emphasis on the role of AhR, whose presence and function in MCs have been recently described [16–19].

## 2. Mast Cells and Microbes

The skin is populated by a rich community of microorganisms in which relatively few taxa, including *Staphylococcus* spp. and the genera *Corynebacterium* and *Propionibacterium*, represent more than 60% of the microbiome [20]. The skin microbiome regulates local immunity by inducing keratinocytes to release antimicrobial peptides and modulating innate and adaptive immune cells, as recently reviewed [20]. Interestingly, the skin microbiome plays an essential role in the maturation and activation of MCs [21, 22]. Specifically, germ-free (GF) mice express low amounts of SCF, and MCs located at the dermis, while numerically unaffected, were phenotypically undifferentiated, with low expression of c-kit [22]. The authors provided a mechanistic explanation of the cross-talk between the microbiome and MCs by showing that staphylococcal lipoteichoic acid (LTA) induced SCF production by epidermal keratinocytes that, in turn, influenced MC maturation [22]. Interestingly, LTA from skin commensal bacteria can also directly influence MC activation by promoting their antimicrobial activity against vaccinia virus by means of production of cathelicidin [21].

While a healthy microbiome is beneficial for MC homeostasis and protective functions, an alteration in the microbiome may promote an aberrant activation of MC with pathological consequences. Atopic dermatitis (AD), a chronic inflammatory cell disease, is associated with an alteration of the skin microbiome with the presence of *S. aureus* colonization, normally absent in healthy skin [20]. While the causative role of *S. aureus* in AD is still debated, its presence in lesional skin is associated with disease severity and disease flares [20]. Interestingly, among the virulence factors of *S. aureus* associated with AD, *δ*-toxin, a phenol-soluble modulin peptide, induces the degranulation of MCs and allergic skin disease [23]. Conversely, an aberrant activation of MCs may alter the normal cooperation with commensal bacteria and result in inflammation, as demonstrated in a mouse model of cryopyrin-associated periodic syndromes bearing a missense NLRP3 mutation [24]. Specifically, upon exposure to commensal bacteria, resident MCs expressing the mutated NLRP3 produce TNF*α* that leads to IL-1*β* secretion by MCs, in turn priming additional innate immune cells to produce and amplify IL-1*β* production and inflammation [24]. Thus, the microbiota and MCs initiate cellular events leading to dysregulated IL-1*β* production and skin inflammation in neonatal mice with Nlrp3 mutation. It is possible that MC-mediated mechanisms observed in mutant mice also contribute to the development of IL-1*β*-driven diseases in humans. If so, given the important role played by MCs in the physiopathology of chronic inflammatory skin disorders [25], microbiome therapeutics and diagnostics represent great opportunities toward precision and personalized medicine in dermatological disease.

The gut harbors a highly complex microbiome [26, 27] that plays fundamental roles in tissue homeostasis by maintaining the integrity of the mucosal barrier, providing nutrients, protecting against pathogens, and shaping the mucosal immune system [28]. The gut microbiome has been implicated in an ever increasing array of pathological conditions [29]. MCs have an important immunoregulatory function in the gastrointestinal (GI) tract where MC overactivation can lead to gastrointestinal disorders [30, 31]. GI symptoms also occur in 14–85% of patients with systemic mastocytosis [32]. The fact that GI MCs are in intimate contact with the epithelium and nerves suggests that MCs are involved in regulating, among others, mucosal permeability and intestinal barrier function [31, 33, 34]. This explains why MCs are involved in so many different types of GI diseases of not only allergic nature. By playing a central role at the GI barrier, both in health and disease, a bidirectional cross-talk between the gut microbiome and MCs is likely to occur. For instance, the gut microbiome promote the migration of MCs into the intestine by inducing CXCR2 ligands from intestinal epithelial cells in a MyD88-dependent manner [35], a finding consistent with the observation that germ-free mice have lower mast cell densities in the small intestine than normal mice [35]. In addition, commensal bacteria, e.g., nonpathogenic *E. coli* or *Enterococcus faecalis*, restrain MC degranulation potential in response to different stimuli, thus suggesting a modulatory activity of bacteria in MC effector functions [36–39]. However, the secretion of TNF and chemokines as well as the expression of adhesion and antigen-presenting molecules was induced by *E. coli*, suggesting a functional shift toward innate immunity vs IgE-mediated effects [36]. These data further point to the directive role of commensals on MC functional activity. In general, gram-negative bacteria were superior to gram-positive bacteria in the inhibitory effect that apparently occurs by interference with intracellular signaling [39].

Several microbial components have been reported to contribute to the MC-commensal dialogue at the epithelial surface. There is a plethora of stimuli, including TLR ligands and inflammatory products, that can directly affect non-IgE-mediated MC activation [40, 41]. The TLR2 ligands LTA and Pam3CSK4 were indeed shown to downmodulate Fc*ε*RI and reduce degranulation in human pulmonary MCs and MC lines [42, 43]. Of interest, microbial metabolites, such as the short-chain fatty acids (SCFA), produced upon microbial fermentation of dietary fibers [44], and several tryptophan metabolites acting as ligands of AhR (further discussed below), also affect non-IgE-mediated MC functioning. This is consistent with the expression of both the SCFA receptor GPR43 [45] and AhR [19] on intestinal MCs. For example, butyrate suppresses murine MC proliferation and cytokine production through inhibiting histone deacetylase [46], while the tryptophan metabolite, kynurenine, promotes AhR-dependent MC activation [47]. Altogether, it seems that commensal bacteria can modulate the activity of MCs in a context-dependent manner by selectively favoring the activation of distinct effector molecules. Thus, it is likely that an improper cross-talk between the microbiome and MCs may result in pathological alterations.

## 3. Linking Microbes to Immunity via AhR

The global burden of infectious diseases continues to be high despite improvements in medicine and public health. Poorly controlled inflammation, often initiated by infection, may contribute to the progression or exacerbation of numerous chronic infections, suggesting that the outcome of the infection depends on the net result of two forces: resistance to pathogen and resistance to damage caused by the immune response initiated to eliminate the pathogen [48]. Being endowed with the ability to both promote and restrain immune activation, AhR is well positioned to exert pathogen control and disease tolerance during infection. AhR is an environmental sensor of xenobiotic ligands such as environmental pollutants (e.g., dioxin) but also endogenous compounds generated by host, microbiota, and diet [14]. In this regard, metabolic pathways targeting the amino acid tryptophan can lead to a myriad of metabolites, some of which are AhR ligands [49–51]. AhR is expressed by many cell types in the body and therefore exerts pleiotropic effects by integrating different signaling pathways. At the cellular level, AhR regulates both innate and adaptive immune responses by acting on a number of cells that not only include myeloid effector cells but also nonhematopoietic cells, such as endothelial and epithelial cells. It affects Th17 cells, regulatory T cells (Tregs), and group 3 innate lymphoid cells (ILC3s) and induces effector cytokines, such as IL-17, IL-22, and IL-10 [52–54]. Thus, AhR signaling shapes a number of immune responses and this has profound influences on host-commensal and host-pathogen interactions at mucosal surfaces. AhR has been shown to modulate responses to RNA and DNA viruses, as well as to gram-negative and gram-positive bacterial species, protozoa, and fungi [55, 56]. The function of AhR depends on the type of pathogen and the target organ. For instance, AhR-deficient mice are susceptible to bacterial and fungal infections in the gut but not to bacterial pneumonia [55]. Thus, AhR has pathway-specific roles that modulate host defense mechanisms during infection at least when AhR is tested in model high affinity ligands. The importance of AhR-driven changes in host responses to infection includes the potential for environmentally derived AhR ligands to affect host defense mechanisms during infection. However, and similarly important, other AhR ligands including food-derived compounds [57] and host and microbial metabolites [58] as well as drugs [59] may affect host responses to infection. Although it remains to be determined, the actual influence endogenous AhR ligands will have on shaping the host response at the host-microbe interface, deciphering the cellular targets, molecular mechanisms, and ligands by which AhR contributes to host immunity during microbial exposure may lead to new approaches to deliberately alter immune function to treat and prevent infectious diseases.

## 4. Mast Cells and Microbes: The AhR Link

**Figure 1:**
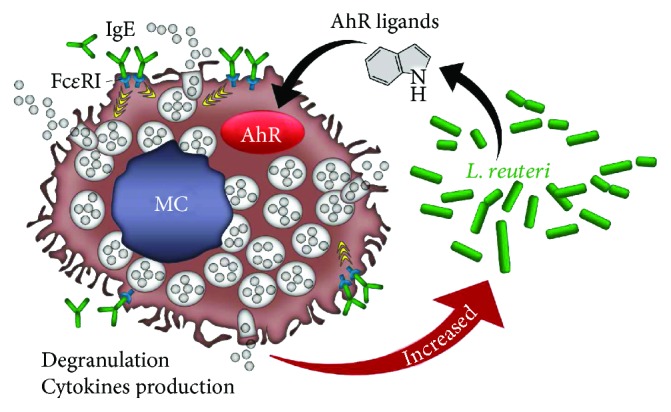
Schematic depiction of the interactions between mast cells and *L. reuteri*. The panel shows that microbial AhR ligands might influence mast cells (MC) activity via AhR. The panel also shows that MCs seem to be required for maintaining the *L. reuteri* pool size, thus supporting the hypothesis of a cross-talk between MCs and *L. reuteri* whereby MCs regulate the abundance of those microbes that produce the AhR ligands that in turn sustain their activity. Details are described in the text.

As said, an important link between MCs and the microbiota involves the AhR, a ligand-activated transcription factor that links environmental stimuli to different functions including detoxification and immune homeostasis [14], although opposing findings were obtained in AhR-deficient mice—in which MCs were both decreased [18] or not [60]. In our hands, growth and differentiation of MCs from bone marrow cultures were impaired in AhR-deficient mice as compared to WT mice, in agreement with Zhou et al. [18]. However, we could not find a reduction of MCs in lymph nodes, as reported by Pilz *et al.* [60] (Zelante T., personal communication). Despite the contrasting results in AhR-deficient mice, which require further investigations to define the exact contribution of AhR in the growth and differentiation of MCs, AhR is known to regulate many functions of MCs [19]. After ligation of the prototypical AhR ligand, 6-formylindolo[3,2-b]carbazole (FICZ), MCs potentiate the classical IgE/Ag-dependent response by enhancing the production of histamine, LTC_4_, reactive oxygen species, and the proinflammatory cytokines IL-6 and IL-13 [16]. However, different MC responses can be achieved depending on the duration of AhR stimulation. Acute AhR stimulation by FICZ boosted proinflammatory features of MC activation, whereas prolonged exposure shifted MCs toward IL-17 production and impaired degranulation [16]. Thus, long-term stimulation of AhR may confer anergy to MCs, thus contributing to the amelioration of allergic manifestations. Thus, both protective and nonprotective responses are originated upon stimulation of the AhR/MC axis *in vivo*, a finding to which the nature of the exogenous or endogenous AhR ligands may greatly contribute. For instance, we have evidence that some endogenous AhR ligands, derived from tryptophan degradation by the microbiota, are endowed with the ability to prime MCs for immune tolerance (Zelante T., unpublished observation). The unique versatility of gut MCs has been recently described in *Candida albicans*/host interaction at mucosal surfaces [61, 62]. We have recently shown that MCs play a key role in the balance between immune pathology and tolerance in the gut promoted by the fungus [63, 64], and multiple mechanisms, including the AhR/indoleamine 2,3-dioxygenase (IDO)1 pathway [65], are exploited in these different effector functions. A possible functional IDO1/AhR-MC activation pathway has recently been described in human endometriosis [66] and chronic rhinosinusitis [67]. However, the unique versatility of MCs at the host/environment interface demands for a plethora of molecular mechanisms behind in health and diseases [68, 69]. Of interest, MCs could affect the microbial composition in the gut. We have found that Firmicutes were expanded and Proteobacteria were decreased in the gut of *Kit^W/W-v^* mice as compared to control mice. Clostridiales and not Lactobacilli, known to activate AhR [51, 70], were expanded in *Kit^W/W-v^* mice, a finding suggesting a limited activation of AhR in condition of MC deficiency. This appeared to be the case, as *L. johnsonii* and not *L. reuteri*, known to produce AhR agonists [51, 71], was expanded in *Kit^W/W-v^* mice and this was associated with limited AhR activation [63]. These results indicate the unique ability of MCs to affect the microbial composition. Intriguingly, the limited expansion of commensals Lactobacilli with known AhR agonist activity, such as *L. reuteri*, in condition of MC deficiency, points to a degree of intimacy between MCs and *L. reuteri*, whereby MCs may favor colonization with AhR-agonistic bacteria to promote optimal AhR signaling at mucosal surfaces (Figure 1).

## 5. Conclusions

Research is increasingly revealing the essential involvement of MCs in normal human biology and in human disease. MCs have nonredundant roles in the host response against pathogens, as they can either promote host resistance to infection or contribute to a dysregulated immune response that can increase host morbidity and mortality [64]. However, the role of MCs may go beyond host defense to encompass regulation of metabolism and inflammation. Furthermore, the range of signals to which MCs respond and react includes signals from the body's microbiota, such that dysfunctional MCs and/or dysbiotic changes may potentially lead to many adverse consequences [72]. The complex interactions between microbiota and MCs demand for more research to better understand and leverage these interactions.
